# Supplementary material for: Heterogeneity of Phenotypic and Functional Changes to Porcine Monocyte-Derived Macrophages Triggered by Diverse Polarizing Factors In Vitro
Source: Int J Mol Sci. 2023 Feb 28;24(5):4671. doi: 10.3390/ijms24054671 (PMC10003195; doi:10.3390/ijms24054671)
Supplement: Supplementary file 1 [file ijms-24-04671-s001.zip › ijms-2213042-supplementary.pdf]

Table S1. Oligonucleotide Primer and Probe sets for detection of several porcine viruses in pig blood.

Virus	Sequences	Reference / Accession
PCV2	F: 5′-TGGCCCGCAGTATTCTGATT-3′<br>R: 5′-CAGCTGGGACAGCAGTTGAG-3′<br>Probe 5′-FAM-CCAGCAATCAGACCCCGTTGGAATG-BHQ1-3′	[Opriessing et al., 2003]
PPV	F: 5′-GAAGACTGGATGATGACAGATCCA-3′<br>R: 5′-TGCTGTTTTTGTTCTTGCTAGAGTAA-3′<br>Probe 5′-VIC-AATGATGGCTCAAACCGGAGGAGA-BHQ1-3′	[Song et al., 2010]
ASFV	F: 5′-CTGCTCATGGTATCAATCTTATCG A-3′<br>R: 5′-GATACCACAAGATCRGCCGT-3′<br>Probe 5′-FAM-CCACGGGAGGAATACCAACCCAGTG-TAMRA-3′	[King et al., 2003]

Table S2. Antibodies used for flow cytometry.

Antibody	Reactivity	Clone	Isotype	Conjugate	Concentration (mg/mL)	Working dilution*
Primary Antibodies						
MHC I	Pig	JM1E3	Mouse IgG1	-	0.1	1/40
CD14	Human**	Tuk4	Mouse IgG2a	Per-CP	ND	1/5
MHC II DR	Pig	2E9/13	Mouse IgG2b	-	0.1	1/25
CD163	Pig	2A10/11	Mouse IgG1	PE	ND	1/4
CD16	Pig	G7	Mouse IgG1	PE	ND	1/4
CD169	Pig	3B11/11	Mouse IgG1	FITC	0.1	1/4
Secondary Antibodies						
Anti-IgG1	Mouse	A85-1	Rat IgG1	BV421	0.2	1/25
Anti-IgG2b	Mouse	R12-3	Rat IgG2a	BV786	0.2	1/25

ND: not determined. * 10 μL of diluted antibody were added to cell pellets (100 μL total).

** Cross-reactive with pig [Jacobsen et al., 1993]

Table S3. Gene list for pig cytokines analyzed through PCR array.

Position	GenBank	Symbol	Description
A01	NM_214370	ADIPOQ	Adiponectin, C1Q and collagen domain containing
A02	NM_213876	AMCF-II	Alveolar macrophage-derived chemotactic factor-II
A03	NM_001195399	BMP2	Bone morphogenetic protein 2
A04	NM_001206388	BMP3	Bone morphogenetic protein 3
A05	NM_001101031	BMP4	Bone morphogenetic protein 4
A06	NM_001168001	BMP6	Bone morphogenetic protein 6
A07	NM_001105290	BMP7	Bone morphogenetic protein 7
A08	NM_001001646	C5	Complement component 5
A09	NM_001166491	CCL1	Chemokine (C-C motif) ligand 1
A10	NM_001256774	CCL11	CCL11
A11	NM_001256147	CCL17	Chemokine ligand 17-like protein
A12	NM_001170516	CCL19	Chemokine (C-C motif) ligand 19
B01	NM_214214	CCL2	Chemokine (C-C motif) ligand 2
B02	NM_001024589	CCL20	Chemokine (C-C motif) ligand 20
B03	NM_001005151	CCL21	Chemokine (C-C motif) ligand 21
B04	NM_001256776	CCL22	C-C motif chemokine 22-like
B05	NM_001025214	CCL25	Chemokine (C-C motif) ligand 25
B06	NM_001003922	CCL27	Chemokine (C-C motif) ligand 27
B07	NM_001024695	CCL28	Chemokine (C-C motif) ligand 28
B08	NM_001009579	CCL3L1	Chemokine (C-C motif) ligand 3-like 1
B09	NM_213779	CCL4	Chemokine (C-C motif) ligand 4
B10	NM_001129946	CCL5	Chemokine (C-C motif) ligand 5
B11	NM_001164515	CCL8	Chemokine (C-C motif) ligand 8
B12	NM_214126	CD40LG	CD40 ligand
C01	NM_001244523	CSF1	Colony stimulating factor 1 (macrophage)
C02	NM_214118	CSF2	Colony stimulating factor 2 (granulocyte-macrophage)
C03	NM_213842	CSF3	Colony stimulating factor 3 (granulocyte)
C04	NM_001008691	CXCL10	Chemokine (C-X-C motif) ligand 10
C05	NM_001128491	CXCL11	Chemokine (C-X-C motif) ligand 11
C06	NM_001009580	CXCL12	Chemokine (C-X-C motif) ligand 12
C07	XM_005652553	LOC396594	Growth-regulated protein homolog gamma
C08	NM_001114289	CXCL9	Chemokine (C-X-C motif) ligand 9
C09	NM_213806	FASLG	Fas ligand (TNF superfamily, member 6)
C10	NM_001166319	IFN-ALPHA-4	Interferon-alpha-4
C11	NM_001164860	IFN-ALPHA-5	Interferon, alpha 5
C12	NM_001003923	IFNB1	Interferon beta

D01	NM_213948	IFNG	Interferon-gamma
D02	NM_214041	IL10	Interleukin 10
D03	NM_213993	IL12A	Interleukin 12A (natural killer cell stimulatory factor 1, cytotoxic lymphocyte maturation factor 1, p35)
D04	NM_214013	IL12B	Interleukin 12B (natural killer cell stimulatory factor 2, cytotoxic lymphocyte maturation factor 2, p40)
D05	NM_213803	IL13	Interleukin 13
D06	NM_214390	IL15	Interleukin 15
D07	NM_213751	IL16	Interleukin 16
D08	NM_001005729	IL17A	Interleukin 17A
D09	XM_001924366	IL17F	Interleukin 17F
D10	NM_213997	IL18	Interleukin 18 (interferon-gamma-inducing factor)
D11	NM_214029	IL1A	Interleukin 1, alpha
D12	NM_214055	IL1B	Interleukin 1, beta
E01	NM_213861	IL2	Interleukin 2
E02	NM_214415	IL21	Interleukin 21
E03	XM_001926156	IL22	Interleukin 22
E04	NM_001130236	IL23A	Interleukin 23, alpha subunit p19
E05	NM_001007520	IL27	Interleukin 27
E06	NM_214123	IL4	Interleukin 4
E07	NM_214205	IL5	Interleukin 5
E08	NM_214399	IL6	Interleukin 6 (interferon, beta 2)
E09	NM_214135	IL7	Interleukin 7
E10	NM_213867	CXCL8	Interleukin 8
E11	NM_001166043	IL9	Interleukin 9
E12	NM_214189	INHA	Inhibin, alpha
F01	NM_214028	INHBA	Inhibin, beta A
F02	NM_214402	LIF	Leukemia inhibitory factor (cholinergic differentiation factor)
F03	XM_001929161	OSM	Oncostatin-M-like
F04	XM_003131711	LOC100515857	C-C motif chemokine 3-like
F05	XM_003131712	CCL23	C-C motif chemokine 23-like
F06	XM_003131714	CCL16	C-C motif chemokine 16-like
F07	XM_003122707	CNTF	Ciliary neurotrophic factor-like
F08	XM_003129101	CXCL13	C-X-C motif chemokine 13-like
F09	XM_003483282	THPO	Thrombopoietin
F10	XR_305507	LOC100621682	Uncharacterized LOC100621682

F11	XM_003480815	TNFSF9	Tumor necrosis factor ligand superfamily member 9-like
F12	NM_214453	CCL24	Chemokine ligand 24-like protein
G01	NM_214453	LTA	Lymphotoxin alpha (TNF superfamily, member 1)
G02	NM_001185138	LTB	Lymphotoxin beta (TNF superfamily, member 3)
G03	NM_001077213	MIF	Macrophage migration inhibitory factor (glycosylation-inhibiting factor)
G04	NM_214435	MSTN	Myostatin
G05	NM_214023	SPP1	Secreted phosphoprotein 1
G06	NM_214015	TGFB1	Transforming growth factor, beta 1
G07	XM_005653762	TGFB2	Transforming growth factor, beta 2
G08	NM_214022	TNF	Tumor necrosis factor
G09	NM_001024696	TNFSF10	Tumor necrosis factor (ligand) superfamily, member 10
G10	NM_001097498	TNFSF13B	Tumor necrosis factor (ligand) superfamily, member 13b
G11	NM_001025217	TNFSF4	Tumor necrosis factor (ligand) superfamily, member 4
G12	NM_214084	VEGFA	Vascular endothelial growth factor A
H01	XM_003357928	ACTB	Actin, beta
H02	NM_213978	B2M	Beta-2-microglobulin
H03	NM_001206359	GAPDH	Glyceraldehyde-3-phosphate dehydrogenase
H04	NM_001032376	HPRT1	Hypoxanthine phosphoribosyltransferase 1
H05	NM_001244068	RPL13A	Ribosomal protein L13a
H06	SA_00133	SGDC	Pig Genomic DNA Contamination
H07	SA_00104	RTC	Reverse Transcription Control
H08	SA_00104	RTC	Reverse Transcription Control
H09	SA_00104	RTC	Reverse Transcription Control
H10	SA_00103	PPC	Positive PCR Control
H11	SA_00103	PPC	Positive PCR Control
H12	SA_00103	PPC	Positive PCR Control

Table S4. Fold regulation of genes, compared to untreated control (moMΦ), and the corresponding p value.

Symbol	Fold Change (comparing to untreated control)									
	IFN-γ + LPS		IL-4		IL-10		TGF-β		Dexamethasone	
	Fold Change	P value	Fold Change	P value	Fold Change	P value	Fold Change	P value	Fold Change	P value
ADIPOQ	1.41	0.898799	**18.93**	0.245784	**0.26**	0.311849	**0.41**	0.366819	**0.46**	0.451905
AMCF-II	**316.22**	0.086332	1.17	0.962042	0.95	0.747631	**0.21**	0.194714	1.02	0.872972
BMP2	1.46	0.811187	**8.76**	**0.041345**	1.01	0.831411	**7.95**	**0.126749**	1.74	0.581165
BMP3	1.25	0.093870	0.96	0.741109	1.10	0.529871	**2.12**	**0.087463**	0.86	0.585654
BMP4	1.01	0.836705	1.07	0.328594	0.74	0.390419	1.07	0.322555	1.58	**0.006601**
BMP6	1.20	0.228239	0.92	0.566064	1.05	0.748986	1.03	0.836088	0.93	0.968751
Bmp7	1.26	0.080691	0.97	0.785169	1.10	0.496386	1.08	0.484579	0.87	0.608438
C5	0.54	0.146735	0.69	0.293660	0.52	0.202299	1.55	0.264429	0.72	0.298669
CCL1	0.64	0.490723	0.88	0.879924	0.63	0.786665	**0.44**	0.483614	**0.30**	0.629258
CCL11	1.26	0.080691	1.09	0.397541	1.10	0.496386	1.08	0.484579	0.87	0.608438
CCL17	**6.43**	0.371835	**35.56**	0.262220	**0.27**	0.421246	**0.16**	0.330659	**0.38**	0.562640
CCL19	**4.66**	0.108259	0.85	0.934280	0.69	0.720684	0.58	0.625857	0.98	0.986998
CCL2	**14.03**	**0.001259**	0.76	0.653774	**0.43**	0.344959	0.74	0.473345	**0.12**	0.197983
CCL20	**312.89**	0.072047	**0.44**	0.245762	**0.40**	0.210550	**0.43**	0.217928	**0.35**	0.241084
CCL21	**0.04**	0.053818	**0.11**	0.065265	**0.04**	0.053422	**2.82**	0.166626	1.38	0.550709
CCL22	**15.87**	0.169045	**3.62**	0.364137	**0.45**	0.291568	**0.44**	0.312003	**0.48**	0.539619
CCL25	0.94	0.998464	1.16	0.901001	0.72	0.453067	1.13	0.882980	0.90	0.632960
CCL27	0.57	0.442573	0.57	0.510445	0.64	0.370257	0.95	0.812116	0.94	0.950359
CCL28	1.11	0.614539	0.84	0.344777	0.96	0.835452	0.95	0.698848	0.79	0.476183
CCL3L1	**20.76**	0.061944	**4.50**	0.291599	**0.33**	0.210544	**0.41**	0.226104	**0.33**	0.238758
CCL4	**20.12**	0.063459	1.97	0.381615	**0.29**	0.228675	**0.40**	0.241212	**0.22**	0.185621
CCL5	**172.28**	**0.008129**	1.80	0.545869	0.81	0.801215	0.69	0.714567	0.51	0.656894
CCL8	**10.96**	**0.016734**	**3.13**	0.472638	**3.29**	0.450284	**0.25**	0.343964	**0.09**	0.292178
CD40LG	1.75	0.443479	1.15	0.810035	0.99	0.972004	0.67	0.572147	**0.22**	0.229333
CSF1	1.09	0.717084	**0.31**	0.745002	1.16	0.762872	0.68	0.465826	**0.21**	0.077039
CSF2	**23.36**	0.247394	1.14	0.765361	1.49	0.658561	**0.47**	0.346332	**0.47**	0.422733
CSF3	**48.25**	0.124713	0.69	0.484265	0.70	0.342266	0.71	0.408970	0.73	0.538715
CXCL10	**87.19**	**0.026552**	0.79	0.956624	**0.25**	0.382568	**0.22**	0.361102	**0.14**	0.348727
CXCL11	**444.33**	0.107372	0.97	0.753065	**0.15**	0.384130	**0.10**	0.358685	**0.18**	0.385454
CXCL12	1.11	0.983382	0.65	0.372610	1.18	0.865626	0.73	0.440940	0.87	0.905445
LOC396594	**10.55**	**0.046573**	1.19	0.567097	**2.35**	**0.018069**	0.97	0.907300	0.95	0.842801
CXCL9	**197.99**	0.210901	0.92	0.738817	**0.29**	0.488989	**0.19**	0.427839	**0.18**	0.427573
FASLG	**4.84**	0.376693	**2.61**	0.556295	1.03	0.961528	0.73	0.754151	**0.27**	0.493310
IFN-ALPHA-4	0.87	0.780823	0.94	0.786385	0.71	0.961791	0.52	0.615952	0.83	0.949458
IFN-ALPHA-5	**0.48**	0.659246	0.62	0.578156	**0.49**	0.507947	**0.38**	0.412275	0.97	0.862629

IFNB1	**3.01**	0.319459	0.64	0.683864	**0.47**	0.490839	**0.37**	0.277474	0.72	0.671918
IFNG	**5.00**	0.249014	1.41	0.593573	0.68	0.730643	1.24	0.798354	0.81	0.797354
IL10	**6.23**	**0.000323**	0.99	0.996030	1.29	0.129111	0.79	0.268489	1.09	0.616750
IL12A	**7.10**	0.225776	1.92	0.329157	1.60	0.208030	1.08	0.484579	0.89	0.718516
IL12B	**161.04**	0.100413	0.66	0.489125	**0.27**	0.282572	0.52	0.349133	0.52	0.452902
IL13	0.83	0.701606	0.64	0.520049	**0.29**	0.438100	**0.22**	0.275779	0.91	0.969973
IL15	**10.89**	0.058519	0.89	0.802649	0.91	0.720995	**0.26**	**0.048119**	0.55	0.178444
IL16	1.45	0.276810	1.01	0.962666	1.17	0.633706	0.67	0.420192	0.70	0.495230
IL17A	1.26	0.080691	0.97	0.785169	1.10	0.496386	1.08	0.484579	0.87	0.608438
IL17F	**2.68**	0.127199	0.97	0.785169	1.10	0.496386	1.08	0.484579	0.87	0.608438
IL18	**2.78**	0.214693	**3.07**	**0.036076**	**5.68**	**0.013221**	**0.44**	0.160756	**0.16**	0.063833
IL1A	**41.54**	0.099943	1.50	0.510471	**0.34**	0.156483	**0.41**	0.235273	**0.13**	0.081704
IL1B	**290.94**	0.094815	**0.28**	0.362872	**0.39**	0.281574	0.52	0.426182	**0.28**	0.288410
IL2	**2.44**	0.473476	**0.49**	0.481290	0.61	0.572739	**0.46**	0.399657	**0.48**	0.530319
IL21	1.61	0.615785	1.12	0.985394	1.22	0.911951	0.90	0.661645	0.70	0.647184
IL22	**2.21**	0.162350	0.76	0.609335	0.79	0.519216	0.85	0.741712	0.82	0.866219
IL23A	**148.91**	0.207966	0.71	0.490164	0.72	0.499493	0.80	0.604844	1.08	0.943159
IL27	**36.46**	0.197175	**2.06**	0.458069	1.37	0.976053	**0.26**	0.256478	0.68	0.498991
IL4	**2.21**	0.516065	0.97	0.936145	0.82	0.649089	0.64	0.474623	**0.40**	0.431406
IL5	1.66	0.083622	0.98	0.983520	0.69	0.657085	1.06	0.799307	0.50	0.124203
IL6	**121.27**	0.099971	**3.17**	0.312153	1.19	0.157639	1.08	0.484579	0.87	0.608438
IL7	**4.22**	**0.027093**	1.02	0.900724	0.85	0.758511	0.55	0.248570	**0.44**	0.175329
CXCL8	**113.84**	0.050726	0.84	0.740314	**3.58**	0.261363	0.92	0.664966	**0.28**	0.342232
IL9	0.88	0.770579	0.73	0.390911	0.70	0.254055	1.94	**0.042828**	0.54	0.127649
INHA	1.26	0.080691	0.97	0.785169	1.22	0.079380	1.08	0.484579	0.87	0.608438
INHBA	**6.96**	0.082509	1.91	0.403685	0.65	0.400062	0.64	0.309994	**0.24**	0.092909
LIF	**5.53**	0.354193	1.26	0.544941	0.66	0.840076	1.00	0.597938	**0.41**	0.536284
OSM	1.47	0.072695	0.95	0.691821	1.09	0.570445	1.07	0.590724	0.93	0.953109
LOC100515857	**5.72**	0.329887	1.50	0.656379	0.64	0.833237	0.54	0.722551	0.76	0.647228
CCL23	**3.75**	0.065405	0.72	0.540861	0.54	0.228674	0.91	0.625461	**4.94**	**0.008826**
CCL16	**4.91**	0.327274	**2.10**	0.248664	0.91	0.982427	0.66	0.172651	**12.15**	0.064898
CNTF	0.95	0.883670	1.25	0.502325	0.86	0.794505	1.09	0.780425	1.16	0.663864
CXCL13	**107.80**	0.106602	**63.50**	0.183648	**388.82**	0.084008	**0.40**	0.245430	**0.13**	0.130653
THPO	1.26	0.080691	0.97	0.785169	1.10	0.496386	1.08	0.484579	0.87	0.608438
LOC100621682	0.85	0.589436	0.66	0.357561	0.71	0.380193	0.68	0.335024	0.74	0.618856
TNFSF9	1.57	0.403941	0.70	0.537601	0.97	0.928933	0.89	0.884709	0.69	0.354655
CCL24	0.80	0.738930	0.78	0.556617	**0.38**	0.207478	**0.28**	0.136813	0.69	0.532827
LTA	**6.64**	0.381291	1.35	0.831067	0.58	0.613616	**0.30**	0.348546	**0.27**	0.429308
LTB	**4.24**	0.147019	1.31	0.775650	1.29	0.810649	0.52	0.329500	**0.09**	0.157966
MIF	0.96	0.143067	1.05	0.306978	1.07	**0.029596**	0.98	0.570221	0.98	0.398501

MSTN	**4.15**	0.354014	0.63	0.307818	**0.49**	0.257680	0.96	0.729622	1.15	0.926752
SPP1	**2.63**	**0.011572**	**0.45**	0.316671	0.76	0.612483	0.68	0.256444	**0.36**	0.084537
TGFB1	**3.26**	0.059263	1.33	0.466186	1.25	0.529799	1.16	0.637755	0.71	0.499294
TGFB2	**0.40**	**0.030825**	1.05	0.588242	0.63	0.112128	**3.39**	**0.022099**	1.62	0.198610
TNF	**12.06**	0.096566	1.33	0.753960	0.88	0.682404	**0.35**	0.370171	**0.14**	0.324941
TNFSF10	**13.68**	0.060468	**2.10**	0.357214	0.51	0.351975	0.90	0.795521	**0.29**	0.190531
TNFSF13B	1.60	0.305973	1.71	0.188449	**3.79**	**0.002243**	1.20	0.594530	0.80	0.669184
TNFSF4	1.45	0.415392	0.82	0.579792	0.76	0.923997	0.97	0.906253	1.05	0.910737
VEGFA	**6.08**	0.140168	0.80	0.899716	0.64	0.364197	1.28	0.569383	0.64	0.654128

Genes modulated in macrophages stimulated with IFN-γ and LPS compared to control, with foldchange > 2, are highlighted in yellow, whereas those with foldchange < 0.5 are highlighted in light yellow. Genes modulated in macrophages stimulated with IL-4 compared to control, with foldchange > 2, are highlighted in pink, whereas those with foldchange < 0.5 are highlighted in violet. Genes modulated in macrophages stimulated with IL-10 compared to control, with foldchange > 2, are highlighted in blue whereas those with foldchange < 0.5 are highlighted in quite blue. Genes modulated in macrophages stimulated with TGF-β compared to control, with foldchange > 2, are highlighted in green, whereas those with foldchange < 0.5 are highlighted in quite green. Genes modulated in macrophages stimulated with dexamethasone compared to control, with foldchange > 2, are highlighted in grey, whereas those with foldchange < 0.5 are highlighted in light gray.

Table S5. Expression of TLR2 and TLR3 genes in pig moMΦ

blood donor pig	TLR2	TLR3
#2659	8.5	8.7
	8.7	8.3
#2661	8.0	5.8
	7.7	5.3
#2656	7.7	6.9
	8.0	6.8
#2658	8.1	7.4
	8.1	7.3
#2662	6.2	5.1
	6.8	3.9

Gene expression levels of *TLR2* and *TLR3* were determined using qPCR. ΔCq values are presented, which were calculated subtracting those of house-keeping gene. ΔCq = Cq (target gene) − Cq (house-keeping gene).

Table S6. Oligonucleotide Primer Sets for Evagreen qRT Real-Time PCR in pig moMΦ .

Gene	Sequences	Reference/Accession
IL-1β	F: 5′-AATTCGAGTCTGCCCTGTACCC-3′<br>R: 5′-TGGTGAAGTCGGTTATATCTTGGC-3′	[Razzuoli et al., 2017]
IL-1RA	F: 5′-ACCTTCATCCGCTCCGACAG-3′<br>R: 5′-GACCTTGACGGCTGCTTTGG-3′	NM_214262.1
IL-6	F: 5′-CAGAGATTTTGCCGAGGATG-3′	[Carta et al., 2021]

	R: 5′-TGGCTACTGCCTTCCCTACC-3′	
IL-10	F: 5′-AGCCAGCATTAAGTCTGAGAA-3′<br>R: 5′-CCTCTCTTGGAGCTTGCTAA-3′	[Carta et al., 2021]
IL-18	F: 5′- CGTGTTTGAGGATATGCCTGATT -3′<br>R: 5′- TGGTTACTGCCAGACCTCTAGTGA-3′	[Razzuoli et al., 2018]
TNF	F: 5′-TGCCTACTGCACTTCGAGGTTATC-3′<br>R: 5′-CAGATAAGCCCGTCGCCCAC-3′	[Carta et al., 2021]
TGFβ2	F: 5′-AGCGCGATTTGCAGGTATTGA-3′<br>F: 5′-GCCGGTTGGACTGTTGTGAC-3′	XM_021064297.1
CXCL13	F: 5′-CCAACCAGAGAAGTCATAGTCTGGA-3′<br>F: 5′-CAGGTGTCCTTGGTGGGGTT-3′	XM_003129101.3
CCL23	F: 5′-GCTTTCACCGTCCTGCTGAC-3′<br>F: 5′-GCTTTCACCGTCCTGCTGAC-3′	XM_003131712.6
TLR2	5′-CGGCTTCCAAGGATGGAGAAA-3′<br>5′-TCCAGAGAFTTGACCTTGCAG-3′	[Franzoni et al., 2022]
TLR3	5′-TGAAGAACTTGATTTCCTTGGCA-3′<br>5′-GGCATGAAAACACCCTGGAG-3′	[Franzoni et al., 2021]
GAPDH	F: 5′-ACCCAGAAGACTGTGGATGG-3′<br>R: 5′-ACGCCTGCTTCACCACCTTC-3′	[Zevini et al., 2017]

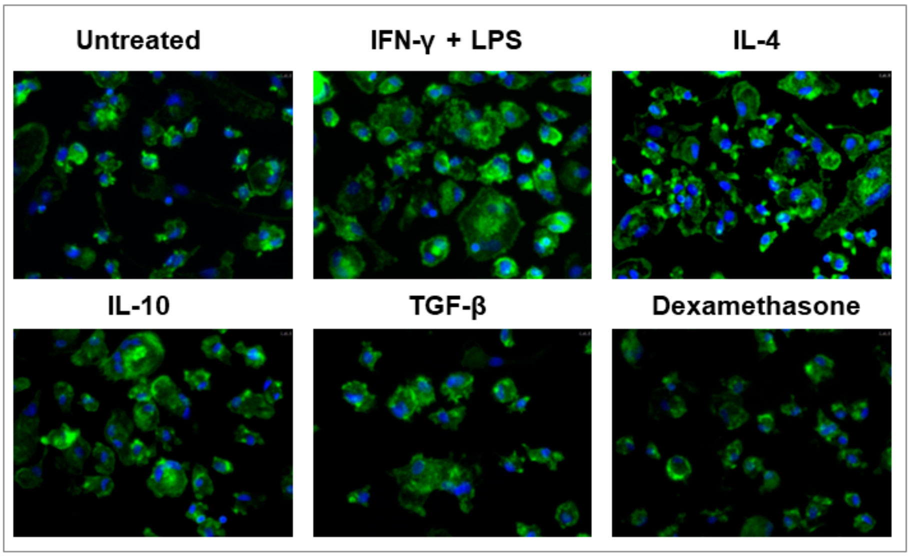

Figure S1. Fluorescent microscopy images of porcine macrophage subsets. Porcine moMΦ were left untreated or stimulated with diverse polarizing factors: IFN-γ and LPS (both at 100 ng/mL), IL-4 (20 ng/mL), IL-10 (20 ng/mL), TGF-β (20 ng/mL), dexamethasone (20 ng/mL). 24 h post-stimulation, morphology were evaluated using microscopy. Fluorescent microscopy images were taken after cytoskeleton staining with Alexa Fluor 488-conjugated phalloidin (green) and nuclei staining with Hoechst 33342 (blue), using an fluorescent inverted stereo microscope (Olympus IX 70, Segrate, Italy) with magnification 40X. Scale bar, 10 μm. Images of six representative macrophage subsets, one from each condition (untreated, IFN- + LPS, IL-4, IL-10, TGF-β, dexamethasone) are presented.

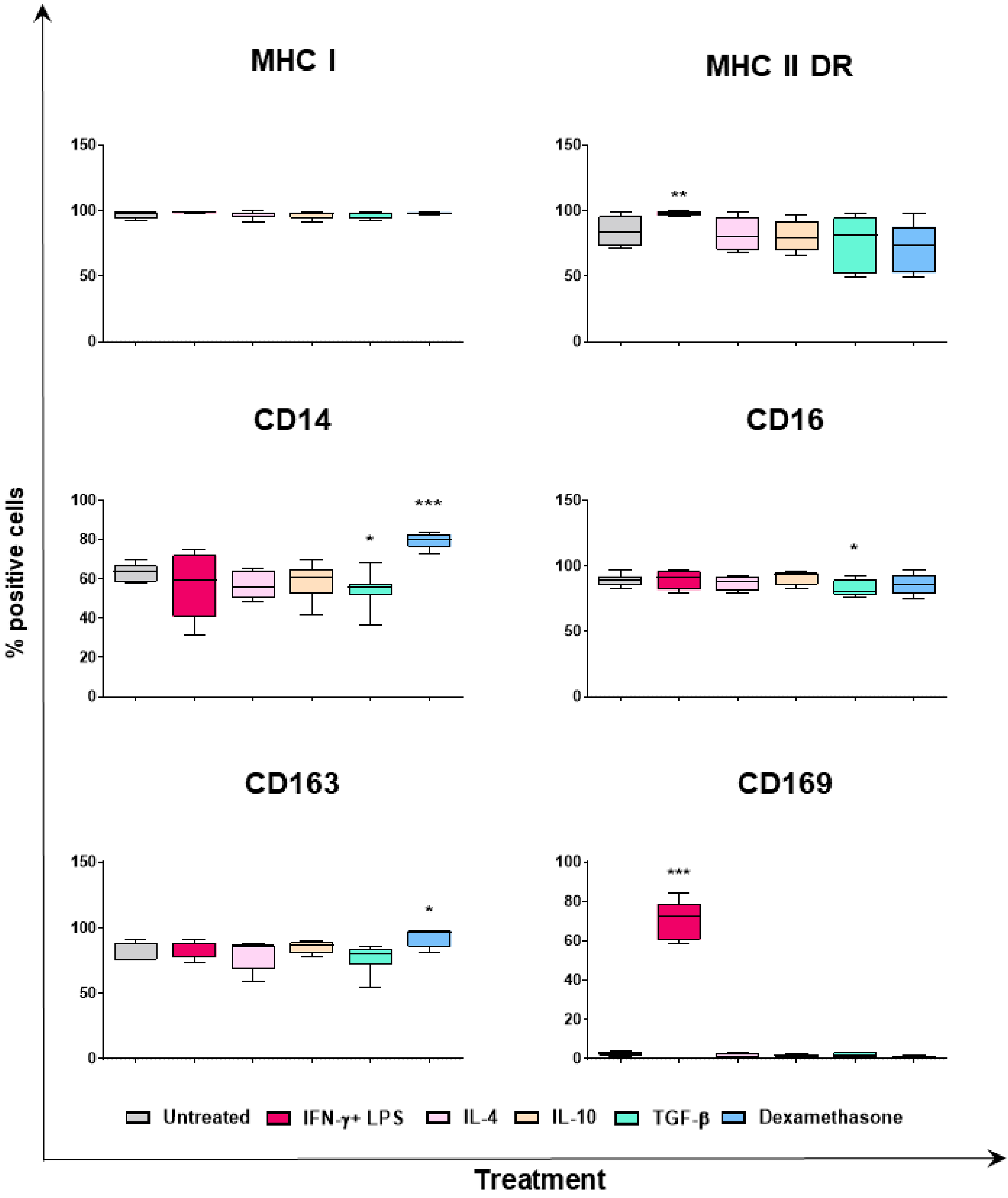

Figure S2. Effect of diverse polarizing factors on porcine moMΦ surface marker expressions (percentages of positive cells). Porcine moMΦ were left untreated or stimulated with diverse polarizing factors: IFN-γ and LPS (both at 100 ng/mL), IL-4 (20 ng/mL), IL-10 (20 ng/mL), TGF-β (20 ng/mL), dexamethasone (20 ng/mL). 24 h post-stimulation, flow cytometry was employed to determine percentages of cells expressing several surface markers: MHC I, MHC II DR, CD14, CD16, CD163, and CD169. Data from three independent experiments utilizing different blood donors are presented. Data are displayed as box-and-whisker plots, showing median and interquartile range (boxes) and minimum and maximum values (whiskers). Values of treated macrophages were compared to the untreated control (moMΦ), using an unpaired T test of a Mann-Whitney test; *** $p < 0.001$, ** $p < 0.01$, * $p < 0.05$.

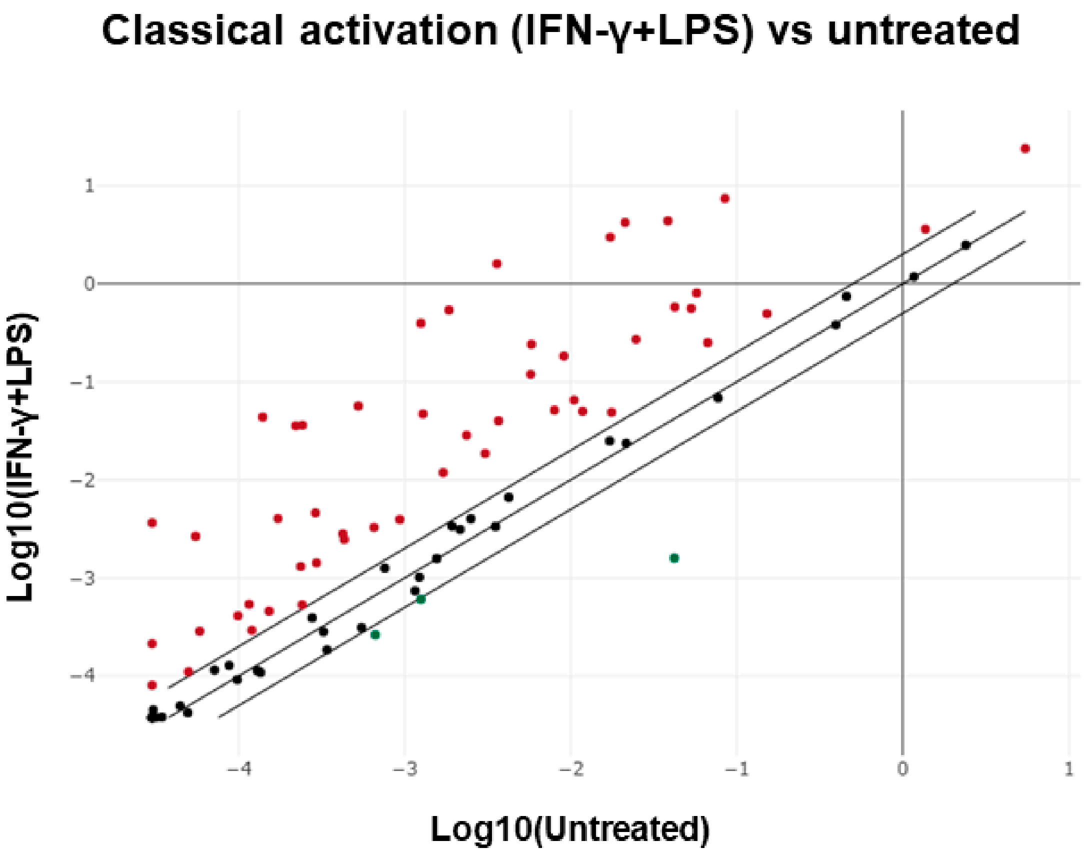

Figure S3. RT 2 Profiler PCR Array analysis of cytokine gene expression: classical activation (IFN-γ + LPS) vs untreated cells. Porcine moMΦ were stimulated with IFN-γ + LPS (both 100 ng/mL) or left untreated. 24h later total RNA was extracted, cDNA was synthesis, and analyzed by RT2 Profiler PCR Array for pig cytokines (PASS-150ZC). Fold changes >2.0 and a P-value < 0.05 were considered to be significant variations. The data represent the mean of three independent experiments using diverse blood donor pigs. Scatter plot on expression levels of 84 porcine cytokine genes are presented. Red dots indicate genes upregulated and green dots indicate gene downregulated in cells stimulated with IFN-γ + LPS compared to untreated controls. The central diagonal line indicates genes that were unchanged, with boundaries representing the two-fold regulation cut-off.

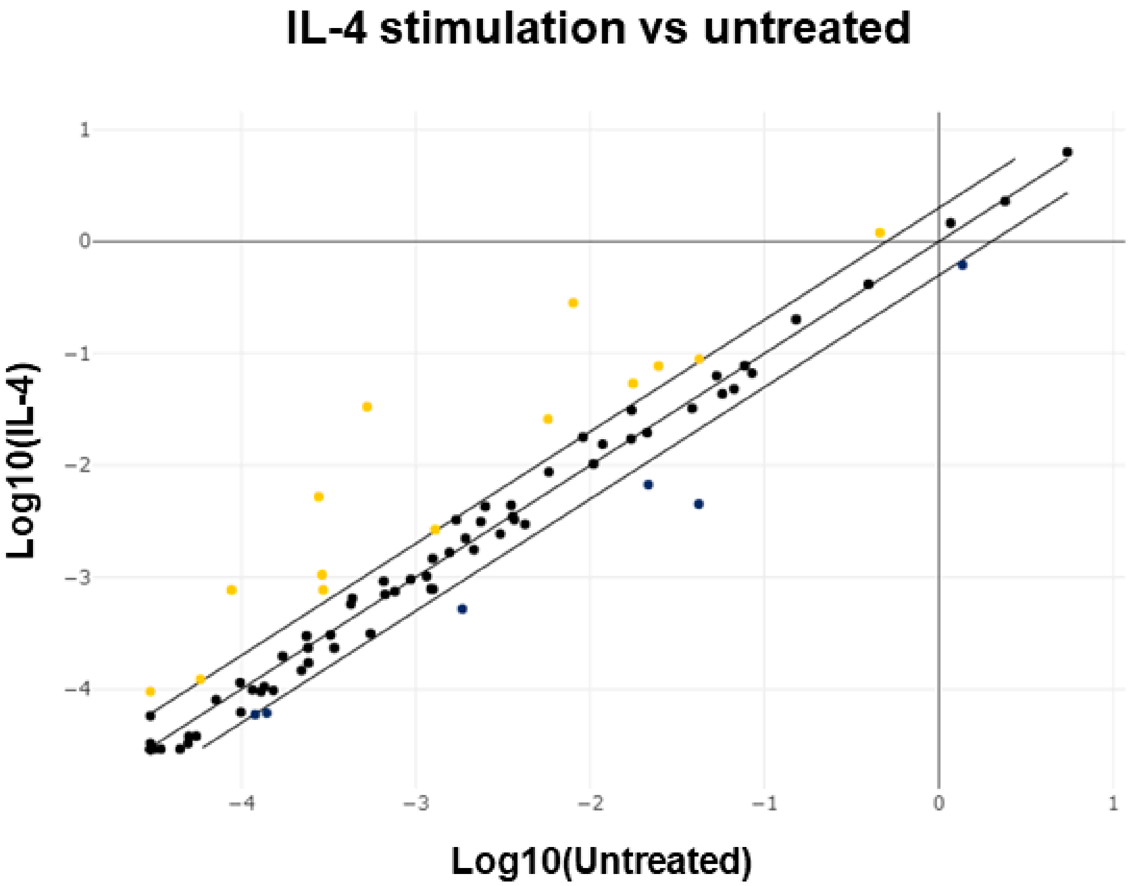

Figure S4. RT 2 Profiler PCR Array analysis of cytokine gene expression: IL-4-stimulation vs untreated cells. Porcine moMΦ were stimulated with IL-4 (20 ng/mL) or left untreated. 24h later total RNA was extracted, cDNA was synthesis, and analyzed by RT2 Profiler PCR Array for pig cytokines (PASS-150ZC). Fold changes >2.0 and a P-value < 0.05 were considered to be significant variations. The data represent the mean of three independent experiments using diverse blood donor pigs. Scatter plot on expression levels of 84 porcine cytokine genes are presented. Yellow dots indicate genes upregulated and blue dots indicate gene downregulated in cells stimulated with IL-4 compared to untreated controls. The central diagonal line indicates genes that were unchanged, with boundaries representing the two-fold regulation cut-off.

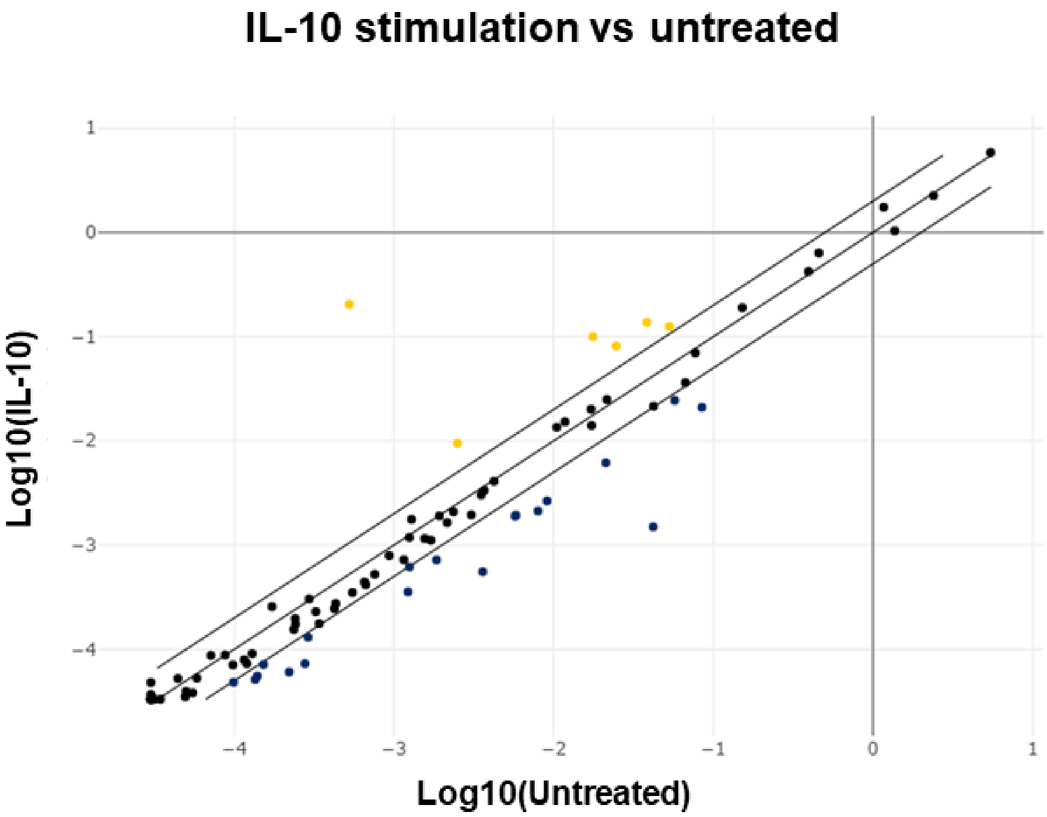

Figure S5. RT 2 Profiler PCR Array analysis of cytokine gene expression: IL-10-stimulation vs untreated cells. Porcine moMΦ were stimulated with IL-10 (20 ng/mL) or left untreated. 24h later total RNA was extracted, cDNA was synthesis, and analyzed by RT2 Profiler PCR Array for pig cytokines (PASS-150ZC). Fold changes >2.0 and a P-value < 0.05 were considered to be significant variations. The data represent the mean of three independent experiments using diverse blood donor pigs. Scatter plot on expression levels of 84 porcine cytokine genes are presented. Yellow dots indicate genes upregulated and blue dots indicate gene downregulated in cells stimulated with IL-10 compared to untreated controls. The central diagonal line indicates genes that were unchanged, with boundaries representing the two-fold regulation cut-off.

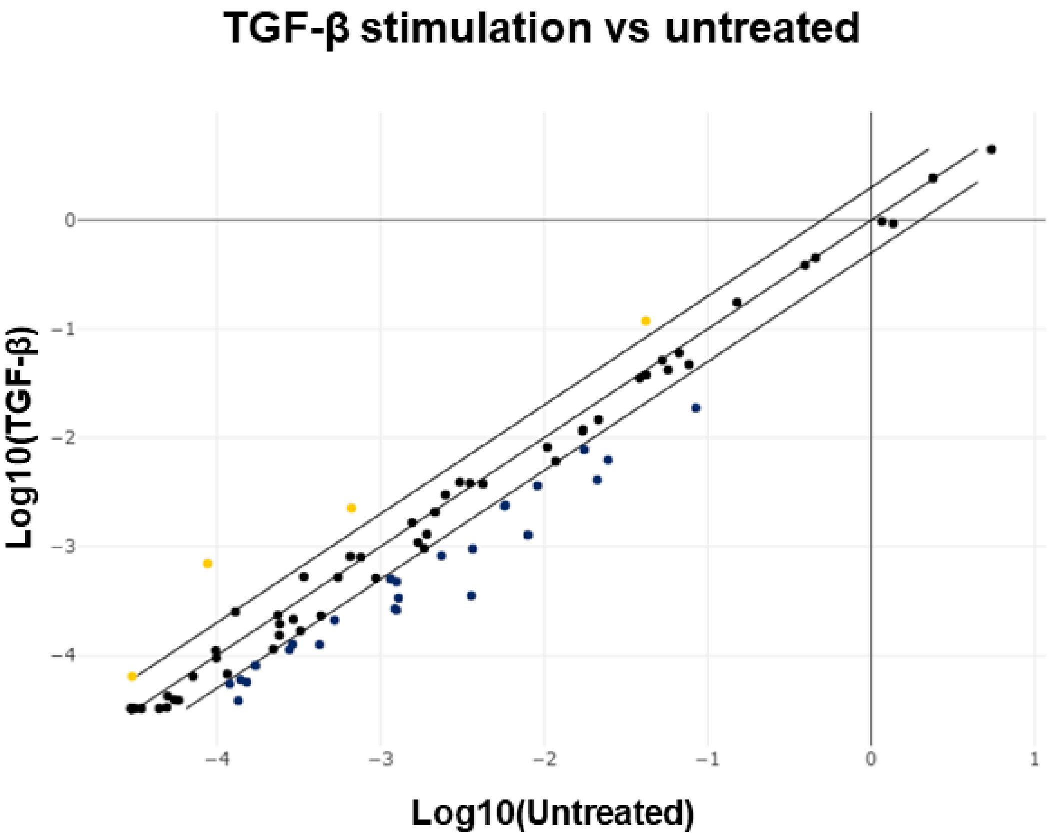

Figure S6. RT 2 Profiler PCR Array analysis of cytokine gene expression: TGF-β-stimulation vs untreated cells. Porcine moMΦ were stimulated with TGF-β (20 ng/mL) or left untreated. 24h later total RNA was extracted, cDNA was synthesis, and analyzed by RT2 Profiler PCR Array for pig cytokines (PASS-150ZC). Fold changes >2.0 and a P-value < 0.05 were considered to be significant variations. The data represent the mean of three independent experiments using diverse blood donor pigs. Scatter plot on expression levels of 84 porcine cytokine genes are presented. Yellow dots indicate genes upregulated and blue dots indicate gene downregulated in cells stimulated with TGF-β compared to untreated controls. The central diagonal line indicates genes that were unchanged, with boundaries representing the two-fold regulation cut-off.

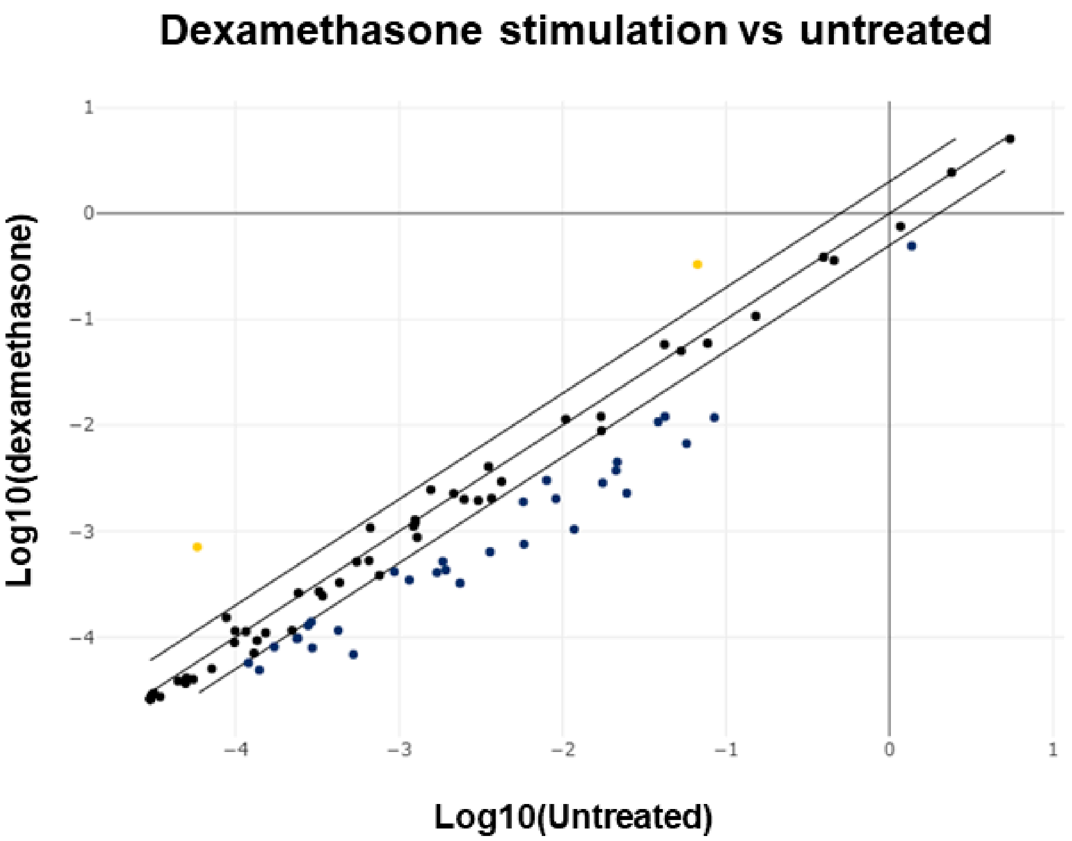

Figure S7. RT 2 Profiler PCR Array analysis of cytokine gene expression: dexamethasone-stimulation vs untreated cells. Porcine moMΦ were stimulated with dexamethasone (20 ng/mL) or left untreated. 24h later total RNA was extracted, cDNA was synthesis, and analyzed by RT2 Profiler PCR Array for pig cytokines (PASS-150ZC). Fold changes >2.0 and a P-value < 0.05 were considered to be significant variations. The data represent the mean of three independent experiments using diverse blood donor pigs. Scatter plot on expression levels of 84 porcine cytokine genes are presented. Yellow dots indicate genes upregulated and blue dots indicate gene downregulated in cells stimulated with dexamethasone compared to untreated controls. The central diagonal line indicates genes that were unchanged, with boundaries representing the two-fold regulation cut-off.

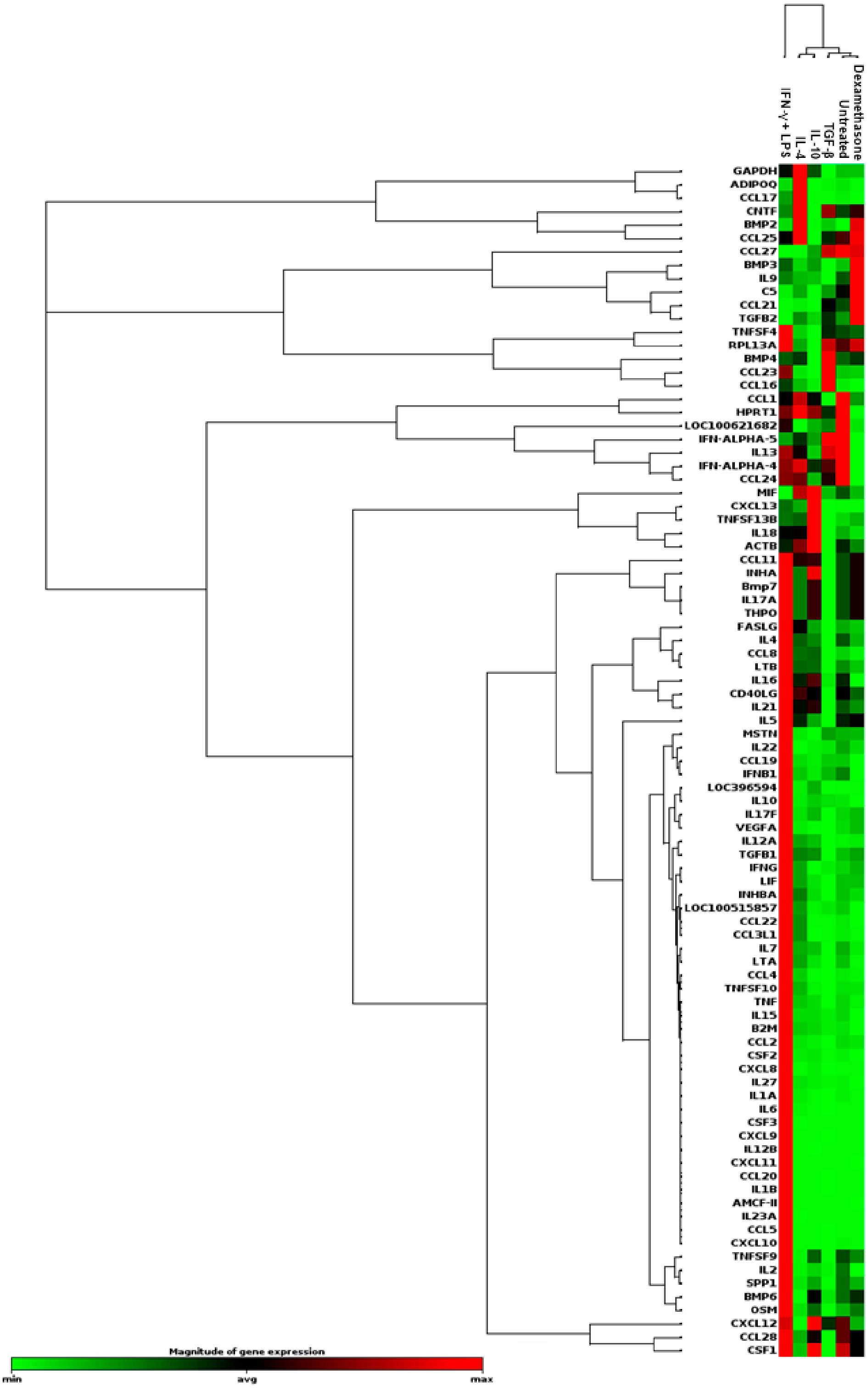

Figure S8. Non-supervised hierarchical clustering analysis of gene expression changes in moMΦ stimulated with diverse polarizing factors. Porcine moMΦ were left untreated or stimulated with diverse polarizing factors: IFN-γ and LPS (both at 100 ng/mL), IL-4 (20 ng/mL), IL-10 (20 ng/mL), TGF-β (20 ng/mL), dexamethasone (20 ng/mL). 24 h post-stimulation, macrophages subsets were analyzed using the RT2 Profiler PCR Array for 84 common immune-related genes. Non-supervised hierarchical clustering analysis of gene expression changes generated using the RT2 Profiler PCR Array for 84 common immune-related genes are presented, based on results obtained in macrophage subsets from three diverse pig blood donors. The colors represent the relative magnitude of gene expression, with brightest red representing the highest value and green representing the smallest value.

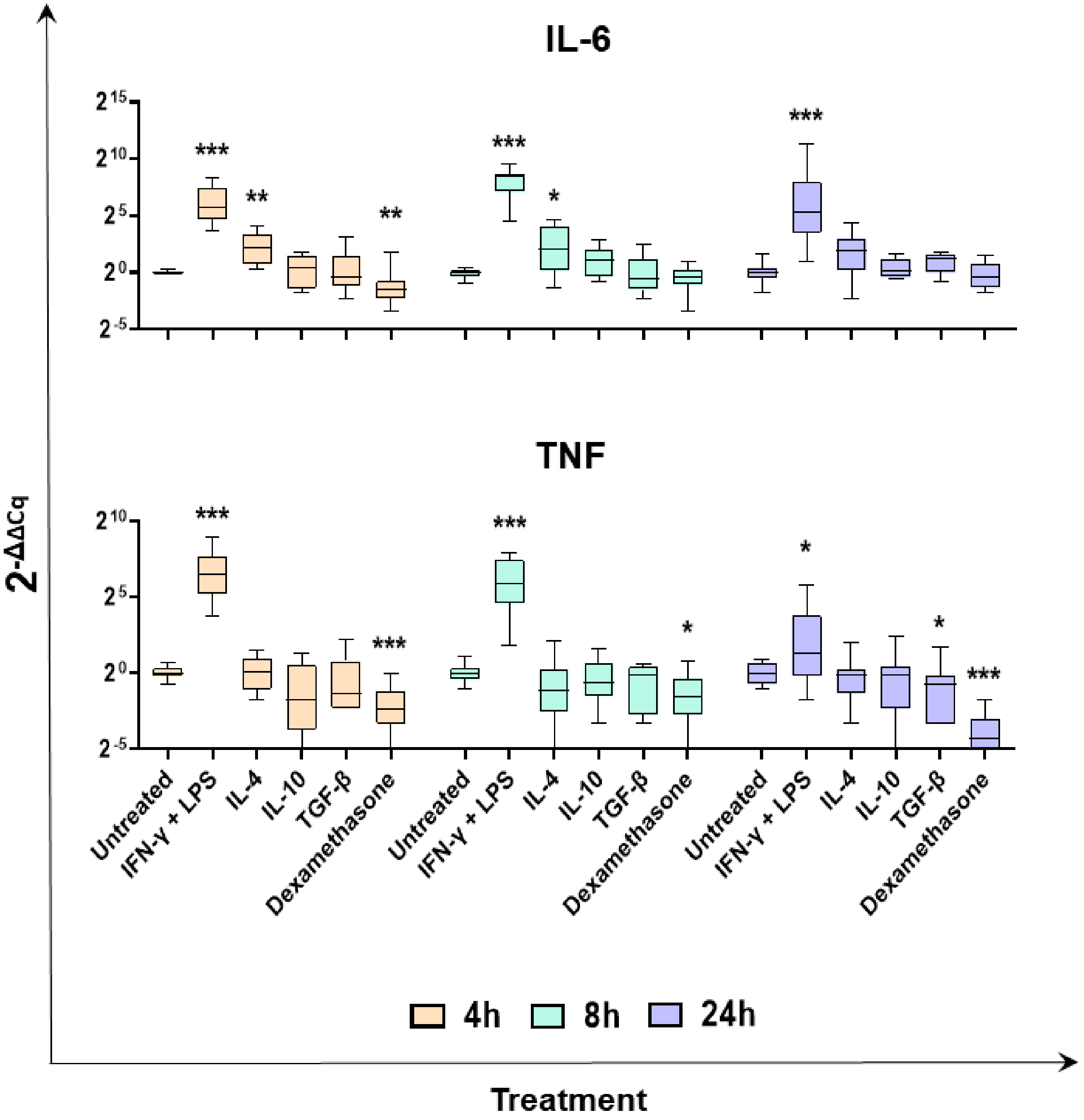

Figure S9. Expression of IL-6 and TNF genes over-time in moMΦ stimulated with diverse polarizing factors. Porcine moMΦ were left untreated or stimulated with diverse polarizing factors: IFN-γ and LPS (both at 100 ng/mL), IL-4 (20 ng/mL), IL-10 (20 ng/mL), TGF-β (20 ng/mL), dexamethasone (20 ng/mL). At 4, 8, and 24 h post-stimulation, gene expression levels of IL-6, TNF were determined using qPCR. At each time point, data were normalized to the values of the untreated control (moMΦ) and expressed as $2^{-\Delta\Delta Cq}$, where $\Delta Cq = Cq$ (target gene) − Cq (house-keeping gene), and $\Delta\Delta Cq = \Delta Cq$ (stimulated samples) − ΔCq (untreated samples). Data from five independent experiments utilizing different blood donors are presented. Data are displayed as box-and-whisker plots, showing median and interquartile range (boxes) and minimum and maximum values (whiskers). Values of treated macrophages were compared to the untreated control (moMΦ), using a Mann-Whitney test; *** $p < 0.001$, ** $p < 0.01$, * $p < 0.05$.

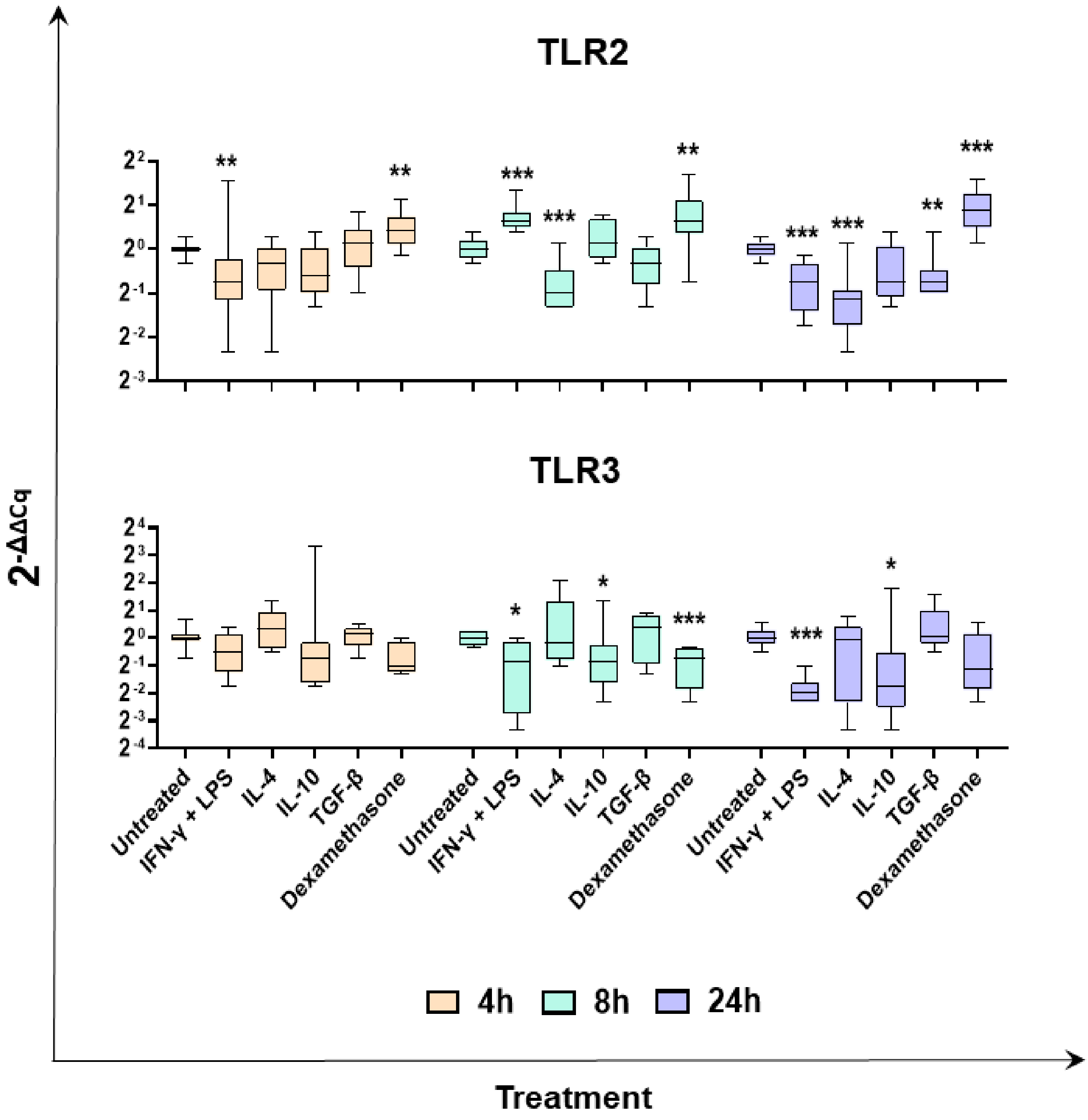

Figure S10. Expression of TLR2 and TLR3 genes over-time in moMΦ stimulated with diverse polarizing factors. Porcine moMΦ were left untreated or stimulated with diverse polarizing factors: IFN-γ and LPS (both at 100 ng/mL), IL-4 (20 ng/mL), IL-10 (20 ng/mL), TGF-β (20 ng/mL), dexamethasone (20 ng/mL). At 4, 8, and 24 h post-stimulation, gene expression levels of IL-6, TNF were determined using qPCR. At each time point, data were normalized to the values of the untreated control (moMΦ) and expressed as 2-ΔΔCq, where ΔCq = Cq (target gene) − Cq (house-keeping gene), and ΔΔCq = ΔCq (stimulated samples) − ΔCq (untreated samples). Data from five independent experiments utilizing different blood donors are presented. Data are displayed as box-and-whisker plots, showing median and interquartile range (boxes) and minimum and maximum values (whiskers). Values of treated macrophages were compared to the untreated control (moMΦ), using a Mann-Whitney test; *** $p < 0.001$, ** $p < 0.01$, * $p < 0.05$.

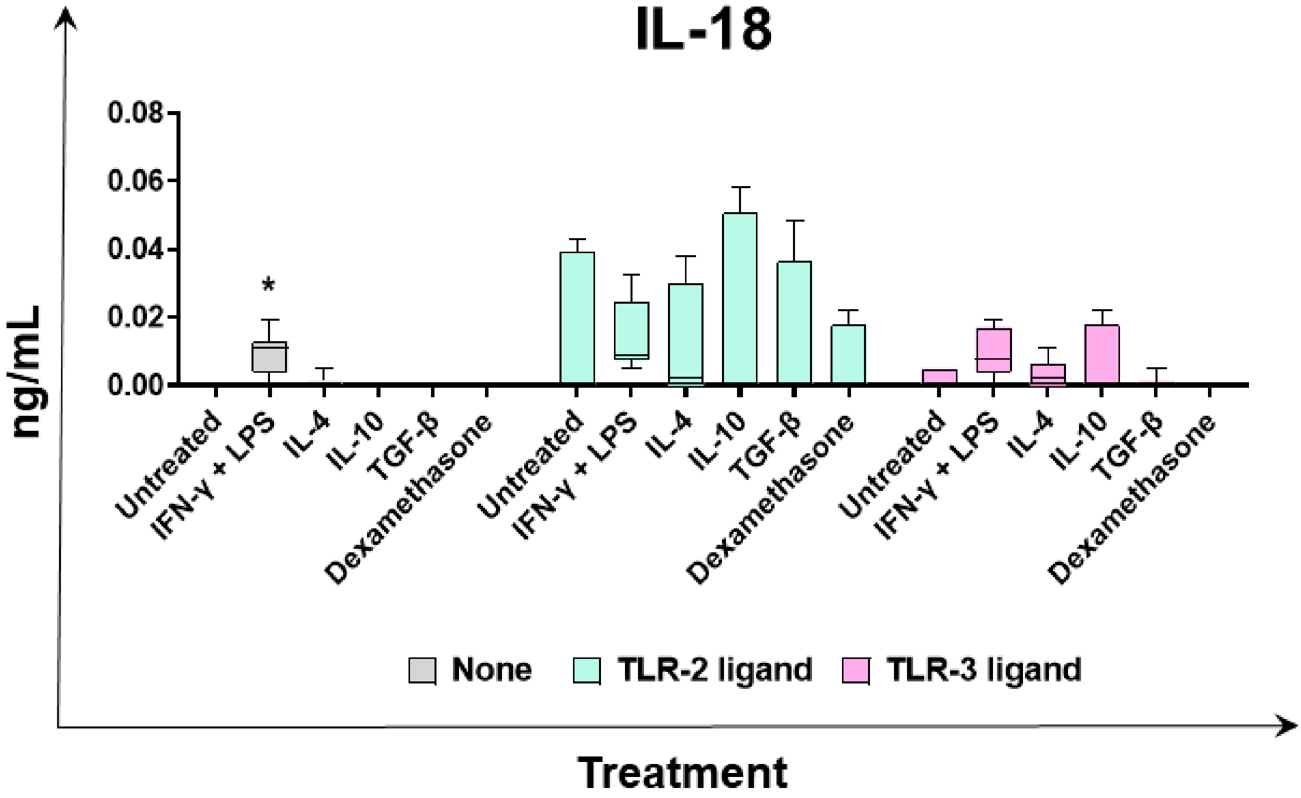

Figure S11. Ability of diverse macrophage subsets to release IL-18 in response to TLR2 or TLR3 agonist stimulation. moMΦ were left untreated or stimulated with diverse polarizing factors: IFN-γ and LPS (both at 100 ng/mL), IL-4 (20 ng/mL), IL-10 (20 ng/mL), TGF-β (20 ng/mL), dexamethasone (20 ng/mL). 24 h later, culture supernatants was replaced with fresh media and cells were left untreated or activated using a TLR-2 ligand (Mag-Pam2Cys_P80; 100 ng/mL) or a TLR-3 ligand (Poly I:C; 100 ng/mL). 24 hours later, the amount of IL-18 in culture supernatants were determined using a multiplex ELISA. Data from three independent experiments utilizing different blood donors are presented. Data are displayed as box-and-whisker plots, showing median and interquartile range (boxes) and minimum and maximum values (whiskers). Values of treated macrophages were compared to the untreated control (moMΦ) using a Mann-Whitney test; * $p < 0.05$.
